# International hypoxia symposium XVIII: 26 February–02 March 2013

**DOI:** 10.1186/2046-7648-2-32

**Published:** 2013-11-14

**Authors:** Matiram Pun, Buddha Basnyat

**Affiliations:** 1 Mountain Medicine Society of Nepal (MMSN), C/O Nepal International Clinic (NIC), Lal durbar, GPO BOX 3596, Kathmandu, Nepal; 2 University of Calgary, 2500 University Dr. NW, AB T2N 1N4, Calgary, Canada; 3 Himalayan Rescue Association, Kathmandu, Nepal; 4 Nepal International Clinic (NIC), Lal Durbar, GPO BOX 3596, Kathmandu, Nepal; 5 Oxford University Clinical Research Unit-Nepal (OCRU-Nepal), Patan Academy of Health Sciences (PAHS), P.O. Box 26500, Kathmandu, Nepal

**Keywords:** Hypoxia, Integrative physiology, Andes, Himalayas

## Abstract

The 18th International Hypoxia Symposia, Lake Louise, Alberta, Canada, February 26–March 02, 2013, covered molecular basis of hypoxic responses (e.g., hypoxia inducible factor, nitrite, nitrate, and hemoglobin) and integrative physiology (e.g., exercise physiology, cerebral blood flow responses, live-high train-low, and population genetics). Free communications and poster sessions covered scientific areas from controlled lab settings to field settings of high altitudes (Andes to Himalayas).

## Background

Hypoxia is an inadequate supply of oxygen to our body which could be generalized (to a whole body) or localized (in a particular tissue). Although slight variation of arterial oxygen concentration in human tissue can be a normal physiology, a significant desaturation for a longer period of time (hypoxia) is a pathological conditi on. Hypoxia occurs in many varied settings, e.g., intensive care units, stroke units, in sleep-disordered breathing, high-altitude excursions, and in cancer tissues. Hypoxia poses a significant threat to the human body and vital organs throughout life. Hence, better understanding of hypoxia pathophysiology is important in various disease management to achieve better outcome. The International Hypoxia Symposia that takes place every 2 years has been a specialized but extremely vibrant conference in the field of hypoxia.

Currently, it is organized by Dr. Robert Roach and Dr. Peter Hackett (both from CO, USA). It was started in 1973 in its earlier form by Dr. Charles Houston. The credit for founding this present day symposia goes to Charles Houston, Geoff Coates and John Sutton. Their vision behind this symposium was to facilitate scientists, clinicians, mountaineers and other interested individuals to share their experiences on lack of oxygen in various settings. The Charlie Houston legacy has been maintained, and the quality of science is excellent. Since 2005, it has always been held at the Chateau at the picturesque Lake Louise in the heart of Banff National Park in the Canadian Rockies during the week of the full moon (February–March of the year).

## Hypoxia XVIII: 2013

The 18th International Hypoxia Symposia started on the evening of the 26th of February, 2013 at Lake Louise, Alberta, Canada inside the luxurious Chateau Lake Louise with a welcome reception. The scientific program on the following days was very exciting with a great mixture of laboratory and field studies.

## 27 February, Wednesday

The first session in the morning was on chronic intermittent hypoxia (CIH), the hallmark of sleep-disordered breathing especially obstructive sleep apnea, and its effect on cardiorespiratory system. The leaders in the field Dr. Nanduri Prabhakar (University of Chicago, Chicago, IL, USA), Dr. Gregg L Semenza and Dr. Larissa Shimoda (both from Johns Hopkins University School of Medicine, Baltimore, MD, USA) covered the topic in great depth. The primary focus was CIH-triggered transcriptional factors, e.g., hypoxia inducible factors (HIF1α and HIF2α), reactive oxygen species (ROS) and their perturbations in cardiorespiratory homeostasis. While controlled experiments with intermittent hypoxia (IH) have promising results to understand physiological effects, they do not exactly reflect what real human patients, e.g., obstructive sleep apnea (OSA), suffer from especially in terms hypercapnia. The experimental limitations were aptly raised by The experimental limitations were aptly raised by Dr. Barbara Morgan (University of Wisconsin-Madison, Madison, WI, USA) and Dr. Prabhakar highlighted the challenges of titrating carbon dioxide in experimental animals. It highlights the need of experimental preparation that addresses true disease picture in human. After coffee break, Dr. Jose Calbet (University of Las Palmas de Gram Canaria, Spain) presented invasive and non-invasive experimental setups in humans to tease out the role of oxygenation (both partial pressure and blood oxygen saturation) during maximal exercise. These sophisticated and invasive experiments are important in understanding human and integrative physiology, but there were few aspects, e.g., role of carbon monoxide in vascular level, that were not adequately elucidated as raised by Dr. Mark Gladwin (University of Pittsburgh, Pittsburgh, PA, USA). Dr. Tatum Simonson (University of California at San Diego, La Jolla, CA, USA) discussed the genetic basis of high-altitude adaptation especially concentrating on a number of publications which emerged in 2010–2012, verifying (genetically) the early observations by Dr. Cynthia Beall from Case Western Reserve University, Cleveland, Ohio, USA (normal range of hemoglobin level among well-adapted Sherpas, Tibetans, and Andeas). Although there is remarkable progress and there are a number of publications in this field, the momentum in search of genetic basis of high adaptation seems slowed down. There are few other aspects, i.e., other markers of adaptations, since it is less likely that they are the only aspects. There has not been much work on the different mechanisms of adaptations between Tibetan vs Andean high-altitude dwellers. Similarly, there are other highlanders from Ethiopia and Kyrgyzstan. We do not know if there are different genetic basis of high adaptations among these groups. The final speaker of the session, Dr. Gladwin (Pittsburgh), discussed the biochemical pathways of nitric oxide (NO) and its intravascular oxidation which subsequently converted it into an anion nitrite which he wonderfully described how it contributes in physiological vasodilation, blood pressure regulation, and cellular responses to stresses like hypoxia and ischemia. The speaker dissected the physiological roles of NO (along with nitrite anion) in vascular and mucosal beds. The experiments and data looked promising for application in clinical trials.

The poster session (I) that day had a number of topics which spanned from ventilatory responses to hypoxia both in animals and humans (Dr. Barbara Morgan, Dr. Jean-Paul Richalet, Dr. Ri-Li Ge, Dr. Marc J Poulin), chemosensitivity to hypoxia in COPDs Dr. Michael K Stickland, Dr. Craig D Steinback), and to high-altitude field studies (from the Himalayas and the Andes) including genetic studies. The Himalayan expedition lead by Dr. Philip N Ainslie (University of British Columbia Okanagan, Kelowna, BC, Canada) and the Andean expedition lead by Dr. Robert Roach (University of Colorado School of Medicine, Aurora, CO, USA) presented interesting physiological experiments from the high-altitude field.

In the evening, Dr. Steve Herrero (University of Calgary, Calgary, AB, Canada) provided an intriguing insight into the life and habitat of bears from the Canadian Rockies. Dr. Herrero who has devoted his life in studying bears highlighted the long and enduring effort of male bears to convince females for mating.

## 28 February, Thursday

The morning session started with Tibetan physiology by Dr. Peter Robbins (University of Oxford, Oxford, UK) who provided an overview of the Tibetan plateau, hypoxia, and inhabitation referring to genomic insights that came out in 2010–2011 which highlighted the adaptive responses to high-altitude hypoxia especially the HIF pathway. He effectively correlated the hypo-responsive HIF pathway to limit excessive red blood cell production, leading to polycythemia. Then, there were some aspects with current globalization and movement, i.e., some of the experimental data among Tibetans who have been living at sea level for a while. The genetic basis of these migrated Tibetans is probably maintained, but the physiological parameters will have to be carefully interpreted since many of them most likely have already spent decades in lowland before they were assessed in Dr. Robbins' physiology lab. Dr. Josef Prchal (University of Utah, Salt Lake City, UT, USA) discussed the hematological aspects of hypoxia-induced transcriptional factors (both up- and down-regulating). Congenital defects and Chuvash polycythemia seem to be perfect examples of hypoxia-sensing mechanism resulting in polycythemia. Dr. Don McClain (University of Utah, Salt Lake City, UT, USA) covered the metabolic adaptation to high attitude. It seems that the adaptation mechanism has led to relative ‘metabolic inflexibility’, i.e., the narrow range in the ability to shift between glucose and fatty acid oxidation. The cardiovascular and hematological parameters are no doubt important aspects of physiological basis of high-altitude adaptation. But at the same time, the data could be useful in understanding other chronic lowland diseases, e.g., Chuvashia polycythemia. After coffee break, ‘The Exercising Hypoxic Brain’ theme began, and Dr. Peter Rasmussen (University of Copenhagen, Copenhagen, Denmark) discussed about exercise-induced hypocapnia leading to decreased cerebral blood flow (CBF) based on the hypothesis that fatigue might be from the central command. Dr. Albert Gjedde (University of Copenhagen, Copenhagen, Denmark) discussed about metabolic demands of the brain and the roles played by glia and neuronal synapses. The final speaker of the session, Dr. Han van Lieshout (Amsterdam Medical Center, Amsterdam, The Netherlands), described the different mechanisms (cardiac diseases, arrhythmias, and orthostatic hypotensions) of syncope and especially about transient global cerebral hypoperfusion relating to different conditions, e.g., hyperventilation (hypocapnia), ice-water immersion, and intense exercise. The session was more of human integrative physiology and primarily focused on exercise physiology. While exercise physiology remains at the center of integrative physiology, it is mandatory that conferences like this should include broader aspects of integrative physiology in the future.

After ski break, the hot topic session (Hot Topics in Hypoxia I) started with the science of looking for key facts about high-altitude adaptation and physiological acclimatization. The roles of nitric oxide and structural features of microcapillary (Dr. Beall) seem important. However, there were quite a large individual variability in the length and tortuosity of microcapillaries. At the same time, the analyses of video images for capillaries were taken from the clearest section of multiple frames and length of videos. Erythropoetin seems to regulate ventilatory responses in a gender-dependent manner (females), while the familiar drug acetazolamide remains a robust tool to explore hypoxic pathophysiology, e.g., cerebral oxygen metabolism (CMRO_2_, Dr. David Dubowitz from University of California at San Diego, San Diego, CA, USA) and central sleep apnea Dr. Keith R Burgess (University of Sydney, Sydney, Australia), with interesting results. The high-dose intravenous infusion of acetazolamide by Dr. Burgess et al. at high altitude to study sleep and brain blood flow was carefully taken by Dr. Luc J. Teppema (Leiden University Medical Centre, Leiden, The Netherlands) and Dr. Jerome Dempsey (University of Wisconsin-Madison, Madison, WI, USA), but the physiological variables and results were quite remarkable in this study. The role of dobutamine (along with acetazolamide) infusion in this study on measured physiological parameters was not adequately highlighted. Studies from the high-altitude field expeditions with invasive techniques and other modern methods continue to explore exercise limitation, brain blood flow, and substrate metabolism (Dr. Ainslie), as well as controlled invasive laboratory studies to explore susceptibilities to acute high-altitude illnesses (Dr. Michael Koehle from Univesity of British Columbia, Vancouver, BC, Canada). Again, it was clear that the hunt for new markers (*S*-adenosylmethionine decarboxylase 1) of hypoxic vascular response continues (Dr. Norbert Weissmann from Universities of Giessen and Marburg Lung Centre, Giessen, Germany), while more mechanistic studies keep exploring key cardiopulmonary features of acclimatization (Dr. Glen E Foster from University of British Columbia Okanagan, Kelowna, BC, Canada; Dr. Andrew Lovering from University of Oregon, Eugene, OR, USA and Dr. Ainslie).

In the evening, Bernadette McDonald, writer and mountain culture consultant (Banff, Canada), gave a fascinating talk on the lives and climbing achievements of Polish climbers in the Himalayas during the communist regime. The post-Second World War economic crisis, skeptic communist regime, and mountaineering had tremendous effect on the life, family, and subsistence of these tough Polish climbers, many of them ended up dying up in the mountains.

## 01 March 2013, Friday

The first session theme ‘Cerebral Blood Flow in Hypoxia: From Early Human Experiments to New Discoveries’ started with Dr. John Severinghaus (University of California, San Francisco, CA, USA) highlighting historical perspective of CBF measurements under hypoxic exposure and ventilatory response. He discussed the role of cerebral spinal fluid pH (logarithm of the reciprocal of hydrogen ion concentration) buffering, CBF during hypoxic hypocapnia, and later experiments clamping CO_2_. Dr. Severinghaus provided a detailed account of early high-altitude studies by a team of anesthesiologists doing spinal fluid analysis and infusion studies. This was followed by Dr. Ainslie (UBC, Okanagan) who concluded that initial increase of CBF is not the risk factor of acute mountain sickness (AMS) (ideally, increased CBF should lead to increased ICP and be the predictor of AMS). Dr. Ainslie elegantly highlighted the mechanism of CBF, although autoregulation aspect was not covered. Then, Dr. Andrew Subudhi (University of Colorado School of Medicine, Aurora, CO, USA) discussed about the limitations of the current techniques of measuring CBF. Dr. Subudhi highlighted that other aspects of blood flow measurements to the brain could be equally important especially carotid and vertebral artery blood flow measurements while we try to understand brain from blood flow point of view. After coffee break, a pro-con session on ‘Is Live-High Train-Low (LHTL) Effective for Improving Sea Level Athletic Performance?’ started with Dr. Benjamin Levine (University of Texas Southwestern Medical Center, Dallas, TX, USA) on the affirmative side and Dr. Carsten Lundby (University of Zurich, Zurich, Switzerland) on the opposition side. Dr. Levine showed elegantly how it works, while Dr. Lundby presented randomized trial data as well as meta-analysis against this concept. Dr. Dempsey (Wisconsin-Madison) and Dr. Poulin (University of Calgary, Calgary, AB, Canada) were moderating the session. Dr. Dempsey presented neutral views from the chair's perspective, basically highlighting how intermittent hypoxia exposure (with reference to obstructive sleep apnea and healthy human models of it) affects cardiovascular system. In the end, both Dr. Levine and Dr. Lundby agreed that the LHTL is a case-by-case situation and depends upon individual response. Over the period of discussion, it seemed that we were quite mixed up with continuous hypoxia vs intermittent hypoxia as well as normobaric hypoxia vs hypobaric hypoxia. Future studies will have to meticulously tease out these different modalities of physiological stimuli and their physiological responses (both in terms of adaptation and maladaptation).

After ski break, poster session (II) was presented, where the topics spanned widely from indoor pollution (Dr. Analisa Cogo from University of Ferrara, Ferrara, Italy) to vascular transcriptional factors from high-altitude pulmonary edema patients (Dr. Ri-Li Ge from Qinghai University Medical College, Xining, Qinghai, China), and from hypoxia and field studies in human, exploring key physiological responses to hypoxia during acclimatization from different established research groups (Dr. Ainslie, Dr. Roach, and Dr. Jim L. Rupert from Univesity of British Columbia, Vancouver, BC, Canada). Similarly, the posters on ventilation, vascular responses, and molecular basis were presented from controlled experiments in humans (Dr. Poulin from Calgary, Dr. Dubowitz from San Diego, Dr. Patrick Levy from Hypoxia Pathophysiology Laboratory, INSERM, Grenoble, France and Dr. Lars I Erikson from Karolinska Institute, Stockholm, Sweden). The science presented was largely molecular with modern development of technology.

In the evening, Dr. Tom Hornbein (University of Washington School of Medicine, Seattle, Washington, USA) talked about his audacious venture in succeeding to climb Mt. Everest. He captivated us all with his presentation. The audience provided a standing ovation for his remarkable adventure, contribution to science, and for this wonderful evening session.

## 02 March 2013, February

Dr. Gladwin (Pittsburg) opened the session with intricate details of nitric oxide pathways (nitrate to nitrite to NO) with recent discoveries in the field challenging traditional dogma. The pathophysiological significance and signaling pathways of nitric oxide in different systems and organelles were provided. The field seems very interesting and may lead a number of clinical trials in coming years especially when it looks promising in patient management. However, an important caveat that still remains unclear is the mode of drug delivery. If oral ingestion is as effective as thought, it will be interesting to see the results.

Dr. Andrew Jones (University of Exeter, Exeter, UK) presented about dietary nitrate supplementation on skeletal muscle oxygenation and energetics from normoxia-hypoxia exercise trials. The calibrated ^31^P-MRS and T2 weighted MRI data showed that the dietary supplementation (beetroot juice rich in nitrate) improves muscle energetics, functional capacity, and faster recovery during exercise in hypoxia. It was a robust science and an excellent presentation. The award winning presentation from Dr. Jones was very well appreciated in the conference partly because beetroot is inexpensive and can be consumed as a dietary supplement. The trials conducted by Dr. Jones were interesting especially when the subjects were well controlled with nitrate-depleted beetroot juice. It really seems a promising field, and further studies from different discipline are expected in the future.

The final session Dr. Michael Joyner (Mayo Clinic, Rochester, MN, USA) covered exercise-induced vasodilation during hypoxia, normoxia, and hyperoxia along with perfusion pressure. Dr. Joyner started his presentation by highlighting how integrative physiology helped reduce patient morbidity and mortality from perioperative to postoperative phases especially due to anesthetic complications. The involvement of α-adrenergic and β-adrenergic mechanisms along with nitric oxide (and also the interaction among them) involved in vascular perfusion during exercise (normoxia, hypoxia, and hyperoxia) were discussed. The human integrative physiology was aptly highlighted.

After coffee break, Dr. Mary Slingo (University of Oxford, Oxford, UK) discussed very clearly but in great detail cellular mechanism of hypoxia sensing from animal models of Chuvash polycythemia and correlated them with the findings from human patients. Dr. Slingo did an excellent job in bringing molecular biology (wet lab bench to be integrated in human physiology and patients with Chuvashia model). The last speaker, Dr. Peter Ratcliffe (University of Oxford, Oxford, UK), discussed the overall cellular mechanisms responsible for hypoxia and stress. The genetic basis of hypoxic stress and its physiological responses were summarized. Most importantly, Dr. Ratcliffe pointed out that not all molecular/genetic pathways are translated into physiological responses, which highlights the importance of integrative physiology. It is important to note that the reductionism approach is not the ultimate of all physiological researches, but rather it is integrative.

After ski break, the hot topics in hypoxia (Hot Topics in Hypoxia II) covered a great mixture of human studies (Dr. Bengt Keyser from University of Geneva, Geneva, Switzerland; Dr. Konrad E Bloch and Dr. Marco Maggiorini from University Hospital of Zurich, Zurich, Switzerland; Dr. Ri-Li Ge; Dr. Justin Lawley from Bangor University, Bangor, Gwynedd, UK) and animal (culture) studies along with acute hypoxia to chronic hypoxia (Dr. Jeff F Dunn from University of Calgary, Calgary, AB, Alberta; Dr. Graham Scott from McMaster University, Hamilton, ON, Canada and Dr. Omolara Ogunshola from (University of Zurich, Zurich, Switzerland). The human studies were mainly from the high-altitude fields and had attempted to study disease pathophysiology. At the same time, the laboratory studies were much more mechanistic, trying to understand basic mechanisms.

Finally, in the evening, there was a sumptuous dinner with award presentations and dance to live music. Everyone had a great time and thanked the organizers, chiefly Robert Roach and Peter Hackett.

## Abbreviations

AMS: Acute mountain sickness; CBF: Cerebral blood flow; CIH: Chronic intermittent hypoxia; CMRO2: Cerebral oxygen metabolism; HIF: Hypoxia inducible factor; IH: Intermittent hypoxia; LHTL: Live-high train-low; NO: Nitric oxide; OSA: Obstructive sleep apnea; ROS: Reactive oxygen species.

## Competing interests

The authors declare that they have no competing interests.

## Authors’ contributions

BB provided conceptual framework for the meeting report and edited the manuscript for the final version. MP prepared and edited the manuscript. MP submitted the manuscript and addressed the editors' and reviewers' comments. Both authors read and approved the final manuscript.

## Authors’ information

MP is a clinician from Nepal and recent master’s graduate from University of Calgary in Mountain Medicine and High Altitude Physiology (MMHAP) program under Prof Marc J Poulin. MP is currently in Nepal and working as a research associate with Mountain Medicine Society of Nepal (MMSN) with Dr. Buddha Basnyat. BB is a world renowned high altitude illness researcher from Nepal and currently he is the president of International Society of Mountain Medicine (ISMM) and MMSN. He has been mentoring a number of Nepalese medical students and young clinicians. He also leads Oxford University Clinical Research Unit- Nepal (OCRU-Nepal) looking into the cause of fever in Nepal.

